# Supplementary material for: Immunoinformatics Analysis of SARS-CoV-2 ORF1ab Polyproteins to Identify Promiscuous and Highly Conserved T-Cell Epitopes to Formulate Vaccine for Indonesia and the World Population
Source: Vaccines (Basel). 2021 Dec 9;9(12):1459. doi: 10.3390/vaccines9121459 (PMC8704007; doi:10.3390/vaccines9121459)
Supplement: Supplementary file 1 [file vaccines-09-01459-s001.zip › vaccines-1469966-supplementary.pdf]

Table S1. Conservancy of predicted CTL epitopes among the SARS-CoV-2 variants. Row 1 contain information about the name of the variants according to the WHO, the pangolin lineage of the variants, and the number of retrieved sequences in the bracket. The duplicated sequences were removed prior to the analysis and the percentage of the conservation was calculated based on the number of the unique sequences only, in which the proportion was shown in the bracket.

Start	Epitope sequence	Alpha B.1.1.7 (158)	Beta B.1.351 (374)	Delta B.1.617.2 (1157)	Eta B.1.525 (436)	Gamma P.1 (9)	Iota B.1.526 (24)	Kappa B.1.617.1 (148)	Lambda C.7 (286)	Mu B.1.621 (18)
**295**	**FMGRIRSVY**	100.00% (111/111)	99.32% (145/146)	98.69% (452/458)	100.00% (174/174)	100.00% (8/8)	100.00% (19/19)	100.00% (95/95)	100.00% (113/113)	100.00% (17/17)
**541**	**RVVRSIFSR**	100.00% (111/111)	100.00% (146/146)	99.56% (456/458)	100.00% (174/174)	100.00% (8/8)	100.00% (19/19)	100.00% (95/95)	100.00% (113/113)	100.00% (17/17)
**611**	**WLTNIFGTV**	100.00% (111/111)	100.00% (146/146)	99.78% (457/458)	100.00% (174/174)	100.00% (8/8)	100.00% (19/19)	100.00% (95/95)	100.00% (113/113)	100.00% (17/17)
**806**	**MVTNNTFTL**	100.00% (111/111)	100.00% (146/146)	100.00% (458/458)	100.00% (174/174)	100.00% (8/8)	100.00% (19/19)	100.00% (95/95)	100.00% (113/113)	100.00% (17/17)
**899**	**WSMATYYLF**	100.00% (111/111)	97.95% (143/146)	99.56% (456/458)	100.00% (174/174)	100.00% (8/8)	94.74% (18/19)	100.00% (95/95)	100.00% (113/113)	100.00% (17/17)
**1055**	**VVVNAANVY**	100.00% (111/111)	99.32% (145/146)	99.13% (454/458)	99.43% (173/174)	100.00% (8/8)	100.00% (19/19)	100.00% (95/95)	99.12% (112/113)	100.00% (17/17)
**1140**	**HEVLLAPLL**	100.00% (111/111)	100.00% (146/146)	100.00% (458/458)	100.00% (174/174)	100.00% (8/8)	100.00% (19/19)	100.00% (95/95)	100.00% (113/113)	100.00% (17/17)
**1247**	**FLTENLLLY**	100.00% (111/111)	100.00% (146/146)	98.69% (452/458)	100.00% (174/174)	100.00% (8/8)	100.00% (19/19)	98.95% (94/95)	100.00% (113/113)	100.00% (17/17)
**1254**	**LYIDINGNL**	100.00% (111/111)	100.00% (146/146)	99.78% (457/458)	100.00% (174/174)	100.00% (8/8)	100.00% (19/19)	100.00% (95/95)	100.00% (113/113)	100.00% (17/17)
**1269**	**LVSDIDITF**	100.00% (111/111)	100.00% (146/146)	99.34% (455/458)	100.00% (174/174)	100.00% (8/8)	100.00% (19/19)	100.00% (95/95)	95.58% (108/113)	100.00% (17/17)
**1366**	**ILGTVSWNL**	100.00% (111/111)	100.00% (146/146)	100.00% (458/458)	100.00% (174/174)	100.00% (8/8)	100.00% (19/19)	100.00% (95/95)	100.00% (113/113)	100.00% (17/17)
**1674**	**YLATALLTL**	99.10% (110/111)	100.00% (146/146)	99.34% (455/458)	100.00% (174/174)	100.00% (8/8)	100.00% (19/19)	100.00% (95/95)	98.23% (111/113)	100.00% (17/17)
**2175**	**LLQLCTFTR**	100.00% (111/111)	99.32% (145/146)	99.78% (457/458)	99.43% (173/174)	100.00% (8/8)	100.00% (19/19)	100.00% (95/95)	97.35% (110/113)	100.00% (17/17)
**2327**	**FLAYILFTR**	98.20% (109/111)	100.00% (146/146)	100.00% (458/458)	100.00% (174/174)	100.00% (8/8)	100.00% (19/19)	100.00% (95/95)	100.00% (113/113)	100.00% (17/17)
**2331**	**ILFTRFFYV**	98.20% (109/111)	100.00% (146/146)	100.00% (458/458)	100.00% (174/174)	100.00% (8/8)	100.00% (19/19)	100.00% (95/95)	100.00% (113/113)	100.00% (17/17)
**2350**	**FSYFAVHFI**	100.00% (111/111)	100.00% (146/146)	99.34% (455/458)	100.00% (174/174)	100.00% (8/8)	100.00% (19/19)	100.00% (95/95)	100.00% (113/113)	100.00% (17/17)
**2597**	**FSSTFNVPM**	100.00% (111/111)	100.00% (146/146)	99.56% (456/458)	99.43% (173/174)	100.00% (8/8)	100.00% (19/19)	100.00% (95/95)	99.12% (112/113)	100.00% (17/17)
**2629**	**LSTFISAAR**	100.00% (111/111)	100.00% (146/146)	100.00% (458/458)	100.00% (174/174)	100.00% (8/8)	100.00% (19/19)	100.00% (95/95)	100.00% (113/113)	100.00% (17/17)
**2784**	**AIFYLITPV**	100.00% (111/111)	100.00% (146/146)	99.78% (457/458)	100.00% (174/174)	87.50% (7/8)	100.00% (19/19)	100.00% (95/95)	100.00% (113/113)	100.00% (17/17)
**2786**	**FYLITPVHV**	100.00% (111/111)	100.00% (146/146)	99.78% (457/458)	100.00% (174/174)	100.00% (8/8)	100.00% (19/19)	100.00% (95/95)	100.00% (113/113)	100.00% (17/17)
**2787**	**YLITPVHVM**	100.00% (111/111)	100.00% (146/146)	99.78% (457/458)	100.00% (174/174)	100.00% (8/8)	100.00% (19/19)	100.00% (95/95)	100.00% (113/113)	100.00% (17/17)
**2883**	**FLPRVFSAV**	100.00% (111/111)	97.95% (143/146)	100.00% (458/458)	100.00% (174/174)	100.00% (8/8)	100.00% (19/19)	98.95% (94/95)	99.12% (112/113)	100.00% (17/17)
**3059**	**LAYYFMRFR**	100.00% (111/111)	100.00% (146/146)	99.56% (456/458)	100.00% (174/174)	100.00% (8/8)	100.00% (19/19)	100.00% (95/95)	100.00% (113/113)	94.12% (16/17)

**3060**	**AYYFMRFRR**	100.00% (111/111)	100.00% (146/146)	99.56% (456/458)	100.00% (174/174)	100.00% (8/8)	100.00% (19/19)	100.00% (95/95)	100.00% (113/113)	94.12% (16/17)
**3076**	**VVAFNTLLF**	100.00% (111/111)	100.00% (146/146)	100.00% (458/458)	100.00% (174/174)	100.00% (8/8)	100.00% (19/19)	100.00% (95/95)	100.00% (113/113)	100.00% (17/17)
**3121**	**FLAHIQWMV**	100.00% (111/111)	100.00% (146/146)	99.78% (457/458)	100.00% (174/174)	100.00% (8/8)	100.00% (19/19)	100.00% (95/95)	100.00% (113/113)	100.00% (17/17)
**3137**	**FWITIAYII**	100.00% (111/111)	98.63% (144/146)	9.61% (44/458)	99.43% (173/174)	100.00% (8/8)	100.00% (19/19)	98.95% (94/95)	100.00% (113/113)	100.00% (17/17)
**3152**	**FYWFFSNYL**	99.10% (110/111)	100.00% (146/146)	100.00% (458/458)	100.00% (174/174)	100.00% (8/8)	100.00% (19/19)	100.00% (95/95)	100.00% (113/113)	100.00% (17/17)
**3466**	**VLAWLYAAV**	100.00% (111/111)	100.00% (146/146)	100.00% (458/458)	100.00% (174/174)	100.00% (8/8)	100.00% (19/19)	100.00% (95/95)	100.00% (113/113)	100.00% (17/17)
**3481**	**FLNRFTTTL**	100.00% (111/111)	100.00% (146/146)	99.78% (457/458)	100.00% (174/174)	100.00% (8/8)	100.00% (19/19)	100.00% (95/95)	100.00% (113/113)	100.00% (17/17)
**3582**	**LLLTILTSL**	100.00% (111/111)	100.00% (146/146)	100.00% (458/458)	100.00% (174/174)	100.00% (8/8)	100.00% (19/19)	100.00% (95/95)	100.00% (113/113)	100.00% (17/17)
**3605**	**LYENAFLPF**	99.10% (110/111)	95.89% (140/146)	98.03% (449/458)	97.13% (169/174)	100.00% (8/8)	100.00% (19/19)	90.53% (86/95)	94.69% (107/113)	94.12% (16/17)
**3652**	**VYMPASWVM**	100.00% (111/111)	99.32% (145/146)	99.78% (457/458)	100.00% (174/174)	100.00% (8/8)	100.00% (19/19)	100.00% (95/95)	99.12% (112/113)	100.00% (17/17)
**3684**	**YASAVVLLI**	100.00% (111/111)	100.00% (146/146)	100.00% (458/458)	100.00% (174/174)	100.00% (8/8)	100.00% (19/19)	100.00% (95/95)	99.12% (112/113)	100.00% (17/17)
**3692**	**ILMTARTVY**	100.00% (111/111)	100.00% (146/146)	99.34% (455/458)	100.00% (174/174)	100.00% (8/8)	100.00% (19/19)	100.00% (95/95)	100.00% (113/113)	100.00% (17/17)
**3752**	**FLARGIVFM**	100.00% (111/111)	99.32% (145/146)	100.00% (458/458)	100.00% (174/174)	100.00% (8/8)	100.00% (19/19)	98.95% (94/95)	100.00% (113/113)	100.00% (17/17)
**4030**	**TMLFTMLRK**	100.00% (111/111)	100.00% (146/146)	100.00% (458/458)	100.00% (174/174)	100.00% (8/8)	100.00% (19/19)	100.00% (95/95)	100.00% (113/113)	100.00% (17/17)
**4265**	**VLSFCAFAV**	100.00% (111/111)	100.00% (146/146)	99.78% (457/458)	100.00% (174/174)	100.00% (8/8)	100.00% (19/19)	100.00% (95/95)	100.00% (113/113)	100.00% (17/17)
**4513**	**YTMADLVYA**	100.00% (111/111)	100.00% (146/146)	100.00% (458/458)	99.43% (173/174)	100.00% (8/8)	94.74% (18/19)	100.00% (95/95)	100.00% (113/113)	100.00% (17/17)
**4656**	**YIKWDLLKY**	100.00% (111/111)	100.00% (146/146)	100.00% (458/458)	100.00% (174/174)	100.00% (8/8)	100.00% (19/19)	100.00% (95/95)	100.00% (113/113)	100.00% (17/17)
**4698**	**ILHCANFNV**	100.00% (111/111)	100.00% (146/146)	100.00% (458/458)	100.00% (174/174)	100.00% (8/8)	100.00% (19/19)	100.00% (95/95)	100.00% (113/113)	100.00% (17/17)
**4723**	**KIFVDGVPF**	100.00% (111/111)	100.00% (146/146)	100.00% (458/458)	100.00% (174/174)	100.00% (8/8)	100.00% (19/19)	100.00% (95/95)	100.00% (113/113)	100.00% (17/17)
**4846**	**YYRYNLPTM**	100.00% (111/111)	98.63% (144/146)	100.00% (458/458)	100.00% (174/174)	100.00% (8/8)	100.00% (19/19)	100.00% (95/95)	100.00% (113/113)	100.00% (17/17)
**4862**	**FVVEVVDKY**	100.00% (111/111)	100.00% (146/146)	100.00% (458/458)	100.00% (174/174)	100.00% (8/8)	100.00% (19/19)	87.37% (83/95)	100.00% (113/113)	100.00% (17/17)
**5024**	**MASLVLARK**	100.00% (111/111)	100.00% (146/146)	100.00% (458/458)	100.00% (174/174)	100.00% (8/8)	100.00% (19/19)	100.00% (95/95)	100.00% (113/113)	100.00% (17/17)
**5132**	**FVNEFYAYL**	99.10% (110/111)	100.00% (146/146)	100.00% (458/458)	100.00% (174/174)	100.00% (8/8)	100.00% (19/19)	100.00% (95/95)	100.00% (113/113)	100.00% (17/17)
**5245**	**LMIERFVSL**	100.00% (111/111)	100.00% (146/146)	100.00% (458/458)	100.00% (174/174)	100.00% (8/8)	100.00% (19/19)	100.00% (95/95)	100.00% (113/113)	100.00% (17/17)
**5247**	**IERFVSLAI**	100.00% (111/111)	100.00% (146/146)	100.00% (458/458)	100.00% (174/174)	100.00% (8/8)	100.00% (19/19)	100.00% (95/95)	100.00% (113/113)	100.00% (17/17)
**5250**	**FVSLAIDAY**	100.00% (111/111)	100.00% (146/146)	100.00% (458/458)	100.00% (174/174)	100.00% (8/8)	100.00% (19/19)	100.00% (95/95)	100.00% (113/113)	100.00% (17/17)
**5273**	**HLYLQYIRK**	100.00% (111/111)	100.00% (146/146)	100.00% (458/458)	100.00% (174/174)	100.00% (8/8)	100.00% (19/19)	100.00% (95/95)	100.00% (113/113)	100.00% (17/17)

**5614**	**FAIGLALYY**	100.00% (111/111)	100.00% (146/146)	99.34% (455/458)	99.43% (173/174)	100.00% (8/8)	100.00% (19/19)	100.00% (95/95)	100.00% (113/113)	100.00% (17/17)
**5678**	**YVFCTVNAL**	100.00% (111/111)	100.00% (146/146)	99.13% (454/458)	100.00% (174/174)	100.00% (8/8)	100.00% (19/19)	100.00% (95/95)	100.00% (113/113)	100.00% (17/17)
**6070**	**FKHLIPLMY**	100.00% (111/111)	100.00% (146/146)	99.13% (454/458)	100.00% (174/174)	100.00% (8/8)	100.00% (19/19)	100.00% (95/95)	100.00% (113/113)	100.00% (17/17)
**6108**	**VLWAHGFEL**	100.00% (111/111)	100.00% (146/146)	100.00% (458/458)	98.85% (172/174)	100.00% (8/8)	100.00% (19/19)	100.00% (95/95)	100.00% (113/113)	100.00% (17/17)
**6506**	**FELWAKRNI**	100.00% (111/111)	100.00% (146/146)	100.00% (458/458)	100.00% (174/174)	100.00% (8/8)	100.00% (19/19)	100.00% (95/95)	100.00% (113/113)	100.00% (17/17)
**6585**	**FRNARNGVL**	100.00% (111/111)	100.00% (146/146)	100.00% (458/458)	100.00% (174/174)	100.00% (8/8)	100.00% (19/19)	100.00% (95/95)	100.00% (113/113)	100.00% (17/17)
**6700**	**HLLIGLAKR**	100.00% (111/111)	100.00% (146/146)	99.78% (457/458)	100.00% (174/174)	100.00% (8/8)	100.00% (19/19)	100.00% (95/95)	100.00% (113/113)	100.00% (17/17)
**6714**	**FELEDFIPM**	98.20% (109/111)	100.00% (146/146)	99.34% (455/458)	100.00% (174/174)	100.00% (8/8)	100.00% (19/19)	100.00% (95/95)	100.00% (113/113)	100.00% (17/17)
**6748**	**LLLDDFVEI**	100.00% (111/111)	100.00% (146/146)	100.00% (458/458)	100.00% (174/174)	100.00% (8/8)	100.00% (19/19)	100.00% (95/95)	100.00% (113/113)	100.00% (17/17)
**6848**	**CQYLNTLTL**	100.00% (111/111)	100.00% (146/146)	100.00% (458/458)	100.00% (174/174)	100.00% (8/8)	100.00% (19/19)	100.00% (95/95)	100.00% (113/113)	100.00% (17/17)
**6850**	**YLNTLTLAV**	100.00% (111/111)	100.00% (146/146)	100.00% (458/458)	100.00% (174/174)	100.00% (8/8)	100.00% (19/19)	100.00% (95/95)	100.00% (113/113)	100.00% (17/17)
**6885**	**WLPTGTLLV**	100.00% (111/111)	100.00% (146/146)	100.00% (458/458)	98.28% (171/174)	100.00% (8/8)	100.00% (19/19)	97.89% (93/95)	100.00% (113/113)	100.00% (17/17)
**6978**	**YKLMGHFAW**	100.00% (111/111)	100.00% (146/146)	100.00% (458/458)	100.00% (174/174)	100.00% (8/8)	100.00% (19/19)	100.00% (95/95)	100.00% (113/113)	100.00% (17/17)
**7019**	**YVMHANYIF**	100.00% (111/111)	100.00% (146/146)	100.00% (458/458)	100.00% (174/174)	100.00% (8/8)	100.00% (19/19)	100.00% (95/95)	100.00% (113/113)	100.00% (17/17)
**7026**	**IFWRNTNPI**	100.00% (111/111)	100.00% (146/146)	100.00% (458/458)	99.43% (173/174)	100.00% (8/8)	100.00% (19/19)	100.00% (95/95)	100.00% (113/113)	100.00% (17/17)

Table S2. Conservancy of predicted HTL epitopes among the SARS-CoV-2 variants. Row 1 contain information about the name of the variants according to the WHO, the pangolin lineage of the variants, and the number of retrieved sequences in the bracket. The duplicated sequences were removed prior to the analysis and the percentage of the conservation was calculated based on the number of the unique sequences only, in which the proportion was shown in the bracket.

Start	Epitope sequence	Alpha B.1.1.7 (158)	Beta B.1.351 (374)	Delta B.1.617.2 (1157)	Eta B.1.525 (436)	Gamma P.1 (9)	Iota B.1.526 (24)	Kappa B.1.617.1 (148)	Lambda C.7 (286)	Mu B.1.621 (18)
**447**	**NDNLLEILQKEKVNI**	99.10% (110/111)	100.00% (146/146)	100.00% (458/458)	100.00% (174/174)	100.00% (8/8)	100.00% (19/19)	100.00% (95/95)	99.12% (112/113)	100.00% (17/17)
**448**	**DNLLEILQKEKVNIN**	99.10% (110/111)	100.00% (146/146)	100.00% (458/458)	100.00% (174/174)	100.00% (8/8)	100.00% (19/19)	100.00% (95/95)	99.12% (112/113)	100.00% (17/17)
**554**	**TAQNSVRVLQKAAIT**	100.00% (111/111)	99.32% (145/146)	98.91% (453/458)	95.98% (167/174)	100.00% (8/8)	100.00% (19/19)	98.95% (94/95)	100.00% (113/113)	100.00% (17/17)
**736**	**PKEIIFLEGETLPTE**	100.00% (111/111)	100.00% (146/146)	99.56% (456/458)	100.00% (174/174)	100.00% (8/8)	100.00% (19/19)	100.00% (95/95)	100.00% (113/113)	100.00% (17/17)
**1054**	**PTVVVNAANVYLKHG**	99.10% (110/111)	99.32% (145/146)	98.69% (452/458)	98.85% (172/174)	100.00% (8/8)	100.00% (19/19)	100.00% (95/95)	98.23% (111/113)	0.00% (0/17)
**1187**	**VSSFLEMKSEKQVEQ**	100.00% (111/111)	98.63% (144/146)	99.78% (457/458)	100.00% (174/174)	0.00% (0/8)	100.00% (19/19)	100.00% (95/95)	99.12% (112/113)	100.00% (17/17)
**1211**	**VKPFITESKPSVEQR**	94.59% (105/111)	100.00% (146/146)	98.91% (453/458)	100.00% (174/174)	87.50% (7/8)	100.00% (19/19)	100.00% (95/95)	100.00% (113/113)	100.00% (17/17)
**1349**	**CKSAFYILPSIISNE**	100.00% (111/111)	100.00% (146/146)	99.56% (456/458)	98.85% (172/174)	100.00% (8/8)	100.00% (19/19)	100.00% (95/95)	99.12% (112/113)	100.00% (17/17)
**1350**	**KSAFYILPSIISNEK**	100.00% (111/111)	100.00% (146/146)	99.56% (456/458)	98.85% (172/174)	100.00% (8/8)	100.00% (19/19)	100.00% (95/95)	99.12% (112/113)	100.00% (17/17)
**1355**	**ILPSIISNEKQEILG**	100.00% (111/111)	100.00% (146/146)	100.00% (458/458)	99.43% (173/174)	100.00% (8/8)	100.00% (19/19)	100.00% (95/95)	100.00% (113/113)	100.00% (17/17)
**1356**	**LPSIISNEKQEILGT**	100.00% (111/111)	100.00% (146/146)	100.00% (458/458)	99.43% (173/174)	100.00% (8/8)	100.00% (19/19)	100.00% (95/95)	100.00% (113/113)	100.00% (17/17)
**1357**	**PSIISNEKQEILGTV**	100.00% (111/111)	100.00% (146/146)	100.00% (458/458)	99.43% (173/174)	100.00% (8/8)	100.00% (19/19)	100.00% (95/95)	100.00% (113/113)	100.00% (17/17)
**2944**	**AYESLRPDTRYVLMD**	100.00% (111/111)	99.32% (145/146)	99.78% (457/458)	100.00% (174/174)	87.50% (7/8)	100.00% (19/19)	100.00% (95/95)	100.00% (113/113)	94.12% (16/17)
**2945**	**YESLRPDTRYVLMDG**	100.00% (111/111)	100.00% (146/146)	99.78% (457/458)	100.00% (174/174)	87.50% (7/8)	100.00% (19/19)	100.00% (95/95)	100.00% (113/113)	88.24% (15/17)
**2958**	**DGSIIQFPNTYLEGS**	100.00% (111/111)	98.63% (144/146)	99.13% (454/458)	100.00% (174/174)	100.00% (8/8)	100.00% (19/19)	100.00% (95/95)	99.12% (112/113)	94.12% (16/17)
**3815**	**VSTQEFRYMNSQGLL**	97.30% (108/111)	96.58% (141/146)	99.13% (454/458)	98.85% (172/174)	100.00% (8/8)	100.00% (19/19)	97.89% (93/95)	99.12% (112/113)	100.00% (17/17)
**3944**	**IASEFSSLPSYAAFA**	100.00% (111/111)	95.89% (140/146)	99.78% (457/458)	100.00% (174/174)	100.00% (8/8)	100.00% (19/19)	98.95% (94/95)	100.00% (113/113)	100.00% (17/17)
**3945**	**ASEFSSLPSYAAFAT**	99.10% (110/111)	95.89% (140/146)	99.78% (457/458)	100.00% (174/174)	100.00% (8/8)	100.00% (19/19)	100.00% (95/95)	100.00% (113/113)	100.00% (17/17)
**3951**	**LPSYAAFATAQEAYE**	99.10% (110/111)	94.52% (138/146)	99.56% (456/458)	98.85% (172/174)	100.00% (8/8)	100.00% (19/19)	100.00% (95/95)	100.00% (113/113)	100.00% (17/17)
**4457**	**LIDSYFVVKRHTFSN**	100.00% (111/111)	99.32% (145/146)	100.00% (458/458)	100.00% (174/174)	100.00% (8/8)	100.00% (19/19)	100.00% (95/95)	100.00% (113/113)	100.00% (17/17)
**4458**	**IDSYFVVKRHTFSNY**	100.00% (111/111)	99.32% (145/146)	100.00% (458/458)	100.00% (174/174)	100.00% (8/8)	100.00% (19/19)	100.00% (95/95)	100.00% (113/113)	100.00% (17/17)
**4560**	**NPDILRVYANLGERV**	100.00% (111/111)	100.00% (146/146)	95.63% (438/458)	100.00% (174/174)	100.00% (8/8)	100.00% (19/19)	100.00% (95/95)	100.00% (113/113)	100.00% (17/17)
**4561**	**PDILRVYANLGERVR**	100.00% (111/111)	100.00% (146/146)	95.63% (438/458)	100.00% (174/174)	100.00% (8/8)	100.00% (19/19)	100.00% (95/95)	100.00% (113/113)	100.00% (17/17)

**4761**	**KELLVYAADPAMHAA**	99.10% (110/111)	100.00% (146/146)	99.78% (457/458)	100.00% (174/174)	100.00% (8/8)	100.00% (19/19)	100.00% (95/95)	100.00% (113/113)	100.00% (17/17)
**4830**	**KHFFFAQDGNAAISD**	100.00% (111/111)	100.00% (146/146)	99.78% (457/458)	99.43% (173/174)	100.00% (8/8)	100.00% (19/19)	100.00% (95/95)	100.00% (113/113)	94.12% (16/17)
**4933**	**QMNLKYAISAKNRAR**	100.00% (111/111)	100.00% (146/146)	99.78% (457/458)	100.00% (174/174)	100.00% (8/8)	100.00% (19/19)	100.00% (95/95)	100.00% (113/113)	100.00% (17/17)
**4934**	**MNLKYAISAKNRART**	100.00% (111/111)	100.00% (146/146)	99.78% (457/458)	100.00% (174/174)	100.00% (8/8)	100.00% (19/19)	100.00% (95/95)	100.00% (113/113)	100.00% (17/17)
**4935**	**NLKYAISAKNRARTV**	100.00% (111/111)	100.00% (146/146)	99.78% (457/458)	100.00% (174/174)	100.00% (8/8)	100.00% (19/19)	100.00% (95/95)	100.00% (113/113)	100.00% (17/17)
**5019**	**PNMLRIMASLVLARK**	100.00% (111/111)	100.00% (146/146)	100.00% (458/458)	100.00% (174/174)	100.00% (8/8)	100.00% (19/19)	100.00% (95/95)	100.00% (113/113)	88.24% (15/17)
**5717**	**AKHYVYIGDPAQLPA**	100.00% (111/111)	100.00% (146/146)	100.00% (458/458)	99.43% (173/174)	100.00% (8/8)	100.00% (19/19)	100.00% (95/95)	100.00% (113/113)	100.00% (17/17)
**5775**	**TVSALVYDNKLKAHK**	82.88% (92/111)	100.00% (146/146)	100.00% (458/458)	100.00% (174/174)	100.00% (8/8)	100.00% (19/19)	100.00% (95/95)	100.00% (113/113)	100.00% (17/17)
**5776**	**VSALVYDNKLKAHKD**	82.88% (92/111)	100.00% (146/146)	100.00% (458/458)	100.00% (174/174)	100.00% (8/8)	100.00% (19/19)	100.00% (95/95)	100.00% (113/113)	100.00% (17/17)
**5777**	**SALVYDNKLKAHKDK**	82.88% (92/111)	100.00% (146/146)	100.00% (458/458)	100.00% (174/174)	100.00% (8/8)	100.00% (19/19)	100.00% (95/95)	100.00% (113/113)	100.00% (17/17)
**5834**	**VFISPYNSQNAVASK**	100.00% (111/111)	100.00% (146/146)	99.78% (457/458)	99.43% (173/174)	100.00% (8/8)	100.00% (19/19)	100.00% (95/95)	100.00% (113/113)	100.00% (17/17)
**6046**	**PTGYVDTPNNTDFSR**	100.00% (111/111)	100.00% (146/146)	100.00% (458/458)	100.00% (174/174)	100.00% (8/8)	100.00% (19/19)	100.00% (95/95)	100.00% (113/113)	94.12% (16/17)
**6454**	**LENVAFNVVNKGHFD**	100.00% (111/111)	99.32% (145/146)	100.00% (458/458)	100.00% (174/174)	100.00% (8/8)	100.00% (19/19)	100.00% (95/95)	100.00% (113/113)	100.00% (17/17)
**6726**	**TVKNYFITDAQTGSS**	99.10% (110/111)	100.00% (146/146)	99.34% (455/458)	100.00% (174/174)	100.00% (8/8)	94.74% (18/19)	100.00% (95/95)	100.00% (113/113)	100.00% (17/17)
**7075**	**KGRLIIRENNRVVIS**	100.00% (111/111)	100.00% (146/146)	100.00% (458/458)	100.00% (174/174)	100.00% (8/8)	100.00% (19/19)	100.00% (95/95)	100.00% (113/113)	100.00% (17/17)
**7076**	**GRLIIRENNRVVISS**	100.00% (111/111)	100.00% (146/146)	100.00% (458/458)	100.00% (174/174)	100.00% (8/8)	100.00% (19/19)	100.00% (95/95)	100.00% (113/113)	100.00% (17/17)
**7077**	**RLIIRENNRVVISSD**	100.00% (111/111)	100.00% (146/146)	100.00% (458/458)	100.00% (174/174)	100.00% (8/8)	100.00% (19/19)	100.00% (95/95)	100.00% (113/113)	100.00% (17/17)

Table S3. Five out of 7 SARS-CoV-2 peptides that match with the human peptides were reported in IEDB as experimentally confirmed by T-cell assay. However, 4 peptides were recognized by T-cells from healthy individuals who have not infected by SARS-CoV-2. This table showing the comparison of the degree of homology of SARS-CoV-2 peptides versus peptides from human proteomes and versus peptides from human common cold coronaviruses (229E, HKU, NL63, and OC43). For homology with HCC, only homology of > 60% was considered.

SARS-CoV-2 Epitope	Immunization	Assay Antigen	MHC Restriction	Assay Description	Homology with human peptides (%)	Homology with human common cold coronavirus (%)
^{2784}**AIFYLITPV**^{2792}	Infectious disease via exposure to SARS-CoV2 (Source Organism)	AIFYLITPV	HLA-A*02:01	biological activity activation Positive	AIFYLITLV (77.8 %)	n.a.
^{5614}**FAIGLALYY**^{5622}	Infectious disease via exposure to SARS-CoV2 (Source Organism)	FAIGLALYY	HLA-C*07:02	multimer/tetramer qualitative binding Positive	SYIGLALYY (77.8%)	HKU LAIGLAVYY (77.8%)<br>NL63 CSIGLGLYY (66.7%)<br>OC43 LAIGLAVFY (66.7%)
	Exposure to endemic/ubiqitious agent Coronavirus (Taxonomic Parent) without evidence for disease	FAIGLALYY	HLA-C*07:02	multimer/tetramer qualitative binding Positive		
^{3684}**YASAVVLLI**^{3692}	Exposure to endemic/ubiqitious agent Coronavirus (Taxonomic Parent) without evidence for disease	YASAVVLLI	HLA-C*06:02	multimer/tetramer qualitative binding Positive	VASAVVLLG (77.8%)	229E YESAVVNGI (66.7%)
^{6748}**LLLDDFVEI**^{6756}	Exposure to endemic/ubiqitious agent Coronavirus (Taxonomic Parent) without evidence for disease	LLLDDFVEI	HLA-A*02:01	ELISPOT IFNg release Positive-High	IALDDFVEI (77.8%)	229E LLLDDFVSV (77.8%)<br>HKU LLLDDFVSI (88.9%)<br>NL63 LLLDDFVTI (88.9%)<br>OC43 ILLDDFVAL (66.7%)
	Infectious disease via exposure to SARS-CoV2 (Source Organism)	LLLDDFVEI	HLA-A*02:01	ELISPOT IFNg release Positive-High		
^{3752}**FLARGIVFM**^{3760}	Infectious disease via exposure to SARS-CoV2 (Source Organism)	FLARGIVFM	HLA-A*02:01	multimer/tetramer qualitative binding Positive	XCARGIVFM (77.8%)	HKU FLQSGIVKM (66.7%)<br>OC43 FLQSGIVKM (66.7%)
	Exposure to endemic/ubiqitious agent Coronavirus (Taxonomic Parent) without evidence for disease	FLARGIVFM	HLA-A*02:01	multimer/tetramer qualitative binding Positive		

Table S4. SARS-CoV-2 peptides that do not have homology match with the human peptides were reported in IEDB as experimentally confirmed by T-cell assay. Majority of these peptides were recognized by T-cells from individuals who were infected by SARS-CoV-2.

Epitope	Immunization	Assay Antigen	MHC Restriction	Assay Description	Homology with human common cold coronavirus (%)
^{5678}YVFCTVNAL^{5686}	No immunization was performed	YVFCTVNAL	HLA class I	biological activity activation Positive	229E YVFSTVNAL (88.9%)<br>HKU YVFTTINAL (77.8%)<br>NL63 YIFSTVNAL (77.8%)<br>OC43 YVFTTINAL (77.8%)
	Infectious disease via exposure to SARS-CoV2 (Source Organism)	YVFCTVNAL	HLA class I	biological activity activation Positive	
^{5245}LMIERFVSL^{5253}	No immunization was performed	LMIERFVSL	HLA class I	biological activity activation Positive	HKU LLIERFVSL (88.9%)<br>OC43 LLIERFVSL (88.9%)
	Exposure to endemic/ubiqitious agent Coronavirus (Taxonomic Parent) without evidence for disease	LMIERFVSL	HLA-A*02:01	multimer/tetramer qualitative binding Positive	
^{2787}YLITPVHVM^{2795}	Exposure to endemic/ubiqitious agent Coronavirus (Taxonomic Parent) without evidence for disease	YLITPVHVM	HLA-C*07:01	multimer/tetramer qualitative binding Positive	n.a.
	Exposure to endemic/ubiqitious agent Coronavirus (Taxonomic Parent) without evidence for disease	YLITPVHVM	HLA-A*02:01	multimer/tetramer qualitative binding Positive	
	Infectious disease via exposure to SARS-CoV2 (Source Organism)	YLITPVHVM	HLA-B*15:01	multimer/tetramer qualitative binding Positive	
	Infectious disease via exposure to SARS-CoV2 (Source Organism)	YLITPVHVM	HLA-A*02:01	biological activity activation Positive	
^{1269}LVSDIDITF^{1277}	Infectious disease via exposure to SARS-CoV2 (Source Organism)	LVSDIDITF	HLA-B*35:01	biological activity activation Positive	n.a.
^{7019}YVMHANYIF^{7027}	Infectious disease via exposure to SARS-CoV2 (Source Organism)	YVMHANYIF	HLA-A*32:01	biological activity activation Positive	HKU NVMHANYLF (77.8%)<br>NL63 NTVHANYIF (66.7%)<br>OC43 NVMHANYLF (77.8%)
^{3692}ILMTARTVY^{3700}	Infectious disease via exposure to SARS-CoV2 (Source Organism)	ILMTARTVY	HLA-B*15:01	multimer/tetramer qualitative binding Positive-Low	n.a.
	No immunization was performed	ILMTARTVY	HLA-B*15:01	multimer/tetramer qualitative binding Positive	
^{1055}VVVNAANVY^{1063}	Infectious disease via exposure to SARS-CoV2 (Source Organism)	VVVNAANVY	HLA-B*35:01	biological activity activation Positive	NL63 FVVNAANEN (66.7%)<br>OC43 VVVNPANGH (66.7%)

^{5024}MASLVLARK^{5032}	Infectious disease via exposure to SARS-CoV2 (Source Organism)	MASLVLARK	HLA-A*68:01	biological activity activation Positive	HKU VSSLVLARK (77.8%) OC43 VSSLVLARK (77.8%)
^{5132}FVNEFYAYL^{5140}	Exposure to endemic/ubiqitious agent Coronavirus (Taxonomic Parent) without evidence for disease	FVNEFYAYL	HLA-A*02:01	multimer/tetramer qualitative binding positive	229E FVDDFYGYL (44.4%) HKU FVNEYYEFL (66.7%)
^{4862}FVVEVVDKY^{4870}	Infectious disease via exposure to SARS-CoV2 (Source Organism)	FVVEVVDKY	HLA-B*15:01	multimer/tetramer qualitative binding positive	HKU FVLEVVYKY (77.8%) OC43 FVLEVVYKY (77.8%)
^{2331}ILFTRFFYV^{2339}	Infectious disease via exposure to SARS-CoV2 (Source Organism)	ILFTRFFYV	HLA-A*02:01	biological activity activation Positive	n.a.
	Infectious disease via exposure to SARS-CoV2 (Source Organism)	ILFTRFFYV	HLA-A*02:01	ICS IFNg release Positive-Low	
	Infectious disease via exposure to SARS-CoV2 (Source Organism)	ILFTRFFYV	HLA-A*02:01	ICS IFNg release Positive-Low	
^{3481}FLNRFTTTL^{3489}	No immunization was performed	FLNRFTTTL	HLA class I	biological activity activation Positive	n.a.
	Infectious disease via exposure to SARS-CoV2 (Source Organism)	FLNRFTTTL	HLA class I	biological activity activation Positive	
	Infectious disease via exposure to SARS-CoV2 (Source Organism)	FLNRFTTTL	HLA class I	biological activity activation Positive	
^{1674}YLATALLTL^{1682}	Infectious disease via exposure to SARS-CoV2 (Source Organism)	YLATALLTL	HLA-A*02:01	biological activity activation Positive	n.a.
	Infectious disease via exposure to SARS-CoV2 (Source Organism)	YLATALLTL	HLA-A*02:01	ELISPOT IFNg release Positive-High	
	Exposure to endemic/ubiqitious agent Coronavirus (Taxonomic Parent) without evidence for disease	YLATALLTL	HLA-A*02:01	ELISPOT IFNg release Positive-High	
	Infectious disease via exposure to SARS-CoV2 (Source Organism)	YLATALLTL	HLA-A*02:01	multimer/tetramer qualitative binding positive	
	Exposure to endemic/ubiqitious agent Coronavirus (Taxonomic Parent) without evidence for disease	YLATALLTL	HLA-A*02:01	multimer/tetramer qualitative binding positive	
^{2883}FLPRVFSAV^{2891}	Infectious disease via exposure to SARS-CoV2 (Source Organism)	FLPRVFSAV	HLA class I	biological activity activation Positive	OC43 FLRVVFSQV (66.7%)

	Documented exposure to SARS-CoV2 (Source Organism) without evidence for disease	FLPRVFSAV	HLA class I	biological activity activation Positive	
	Infectious disease via exposure to SARS-CoV2 (Source Organism)	FLPRVFSAV	HLA-A*02:01	biological activity activation Positive	
	No immunization was performed	FLPRVFSAV	HLA class I	biological activity activation Positive	
	Infectious disease via exposure to SARS-CoV2 (Source Organism)	FLPRVFSAV	HLA class I	biological activity activation Positive	
	Exposure to endemic/ubiqitious agent Coronavirus (Taxonomic Parent) without evidence for disease	FLPRVFSAV	HLA-A*02:01	multimer/tetramer qualitative binding positive	
3466VLAWLYAAV3474	No immunization was performed	VLAWLYAAV	HLA class I	biological activity activation Positive	229E VVAFLYAAI (66.7%)<br>HKU VIAWLYAAI (77.8%)<br>NL63 VVAFLYAAL (66.7%)<br>OC43 FVAWLYAAI (66.7%)
	Infectious disease via exposure to SARS-CoV2 (Source Organism)	VLAWLYAAV	HLA class I	biological activity activation Positive	
4698ILHCANFNV4707	Infectious disease via exposure to SARS-CoV2 (Source Organism)	ILHCANFNV	HLA class I	biological activity activation Positive	229E ILHCSNFNT (77.8%)<br>HKU IIHCANFNI (77.8%)<br>NL63 VIHCANFNT (77.8%)<br>OC43 IIHCANFNI (77.8%)
	No immunization was performed	ILHCANFNV	HLA class I	biological activity activation Positive	
6850YLNTLTLAV6858	No immunization was performed	YLNTLTLAV	HLA class I	biological activity activation Positive	HKU YLNTTTLAV (88.9%)<br>OC43 YLNTTTLAV (88.9%)
	Infectious disease via exposure to SARS-CoV2 (Source Organism)	YLNTLTLAV	HLA class I	biological activity activation Positive	
3121FLAHIQWMV3129	Infectious disease via exposure to SARS-CoV2 (Source Organism)	FLAHIQWMV	HLA-A*02:06	biological activity activation Positive	n.a.
	Infectious disease via exposure to SARS-CoV2 (Source Organism)	FLAHIQWMV	HLA-A*02:01	multimer/tetramer qualitative binding positive	
	Exposure to endemic/ubiqitious agent Coronavirus (Taxonomic Parent) without evidence for disease	FLAHIQWMV	HLA-A*02:01	multimer/tetramer qualitative binding positive	
2786FYLITPVHV2794	Exposure to endemic/ubiqitious agent Coronavirus (Taxonomic Parent) without evidence for disease	FYLITPVHV	HLA-C*07:02	multimer/tetramer qualitative binding positive	n.a.
6108VLWAHGFEL6116	Infectious disease via exposure to SARS-	VLWAHGFEL	HLA class I	biological activity activation Positive	229E VLWAGGLEL (77.8%)

	CoV2 (Source Organism)				NL63 VLWAGSLEL (66.7%) OC43 VTWAANFEL (66.7%)
	Infectious disease via exposure to SARS-CoV2 (Source Organism)	VLWAHGFEL	HLA class I	biological activity activation Positive	
	No immunization was performed	VLWAHGFEL	HLA class I	biological activity activation Positive	
^{3652}VYMPASWVM^{3660}	Infectious disease via exposure to SARS-CoV2 (Source Organism)	VYMPASWVM	HLA-A*24:02	biological activity activation Positive	n.a.
	Infectious disease via exposure to SARS-CoV2 (Source Organism)	VYMPASWVM	HLA-A*24:02	multimer/tetramer qualitative binding positive	
^{3059}LAYYFMRFR^{3067}	Infectious disease via exposure to SARS-CoV2 (Source Organism)	LAYYFMRFR	HLA-A*31:01	biological activity activation Positive	n.a.
^{4862}FVVEVVDKY^{4870}	Infectious disease via exposure to SARS-CoV2 (Source Organism)	FVVEVVDKY	HLA-B*15:01	multimer/tetramer qualitative binding positive	HKU FVLEVVYKY (77.8%) OC43 FVLEVVYKY (77.8%)

Table S5. Toxicity of VC. Toxinpred scan for fragments of 10 amino acid length and predict their toxicity. Only residue 29-48 which is part of the beta defensin adjuvant that contain toxic fragments of 10 mer peptides.

Peptide Sequence	SVM score	Prediction	Hydrophobicity	Hydropathicity	Hydrophilicity	Charge	Mol wt
GIINTLQKYY	-0.66	Non-Toxin	-0.04	-0.18	-0.7	1	1212.57
IINTLQKYYC	-0.39	Non-Toxin	-0.05	0.11	-0.8	1	1258.65
INTLQKYYCR	-0.4	Non-Toxin	-0.3	-0.79	-0.32	2	1301.67
NTLQKYYCRV	-0.48	Non-Toxin	-0.32	-0.82	-0.29	2	1287.64
TLQKYYCRVR	-0.56	Non-Toxin	-0.43	-0.92	-0.01	3	1329.72
LQKYYCRVRG	-0.54	Non-Toxin	-0.4	-0.89	0.03	3	1285.67
QKYYCRVRGG	-0.73	Non-Toxin	-0.44	-1.31	0.21	3	1229.56
KYYCRVRGGR	-0.77	Non-Toxin	-0.54	-1.41	0.49	4	1257.61
YYCRVRGGRC	-0.85	Non-Toxin	-0.43	-0.77	0.09	3	1232.57
YCRVRGGRCA	-0.71	Non-Toxin	-0.41	-0.46	0.27	3	1140.47
CRVRGGRCAV	-0.9	Non-Toxin	-0.35	0.09	0.35	3	1076.43
RVRGGRCAVL	-1.08	Non-Toxin	-0.31	0.22	0.27	3	1086.46
VRGGRCAVLS	-1.01	Non-Toxin	-0.16	0.59	0	2	1017.35
RGGRCAVLSC	-1.02	Non-Toxin	-0.21	0.42	0.05	2	1021.35
GGRCAVLSCL	-0.58	Non-Toxin	0.02	1.25	-0.43	1	978.33
GRCAVLSCLP	-0.6	Non-Toxin	0	1.13	-0.43	1	1018.39
RCAVLSCLPK	-0.63	Non-Toxin	-0.13	0.78	-0.13	2	1089.51
CAVLSCLPKE	-0.68	Non-Toxin	-0.01	0.88	-0.13	0	1062.44
AVLSCLPKEE	-0.71	Non-Toxin	-0.08	0.28	0.27	-1	1088.42
VLSCLPKEEQ	-0.76	Non-Toxin	-0.17	-0.25	0.34	-1	1145.48
LSCLPKEEQI	-0.52	Non-Toxin	-0.15	-0.22	0.31	-1	1159.51
SCLPKEEQIG	-0.43	Non-Toxin	-0.19	-0.64	0.49	-1	1103.4
CLPKEEQIGK	-0.44	Non-Toxin	-0.27	-0.95	0.76	0	1144.5
LPKEEQIGKC	-0.33	Non-Toxin	-0.27	-0.95	0.76	0	1144.5
PKEEQIGKCS	-0.08	Non-Toxin	-0.35	-1.41	0.97	0	1118.41
KEEQIGKCST	-0.16	Non-Toxin	-0.36	-1.32	0.93	0	1122.4
EEQIGKCSTR	-0.07	Non-Toxin	-0.43	-1.38	0.93	0	1150.41
EQIGKCSTRG	-0.13	Non-Toxin	-0.35	-1.07	0.63	1	1078.35
QIGKCSTRGR	0.18	Toxin	-0.47	-1.17	0.63	3	1105.42
IGKCSTRGRK	0.03	Toxin	-0.51	-1.21	0.91	4	1105.46
GKCSTRGRKC	0.19	Toxin	-0.58	-1.41	0.99	4	1095.43
KCSTRGRKCC	1.2	Toxin	-0.59	-1.12	0.89	4	1141.51
CSTRGRKCCR	1.57	Toxin	-0.65	-1.18	0.89	4	1169.52
STRGRKCCRR	1.01	Toxin	-0.83	-1.88	1.29	5	1222.57
TRGRKCCRRK	0.47	Toxin	-0.92	-2.19	1.56	6	1263.67
RGRKCCRRKK	0.18	Toxin	-1.01	-2.51	1.9	7	1290.74
GRKCCRRKKE	0.49	Toxin	-0.9	-2.41	1.9	5	1263.67
RKCCRRKKEA	0.25	Toxin	-0.89	-2.19	1.85	5	1277.69
KCCRRKKEAA	0.66	Toxin	-0.69	-1.56	1.5	4	1192.58
CCRRKKEAAA	-0.02	Non-Toxin	-0.55	-0.99	1.15	3	1135.48
CRRKKEAAAK	-0.97	Non-Toxin	-0.67	-1.63	1.55	4	1160.52
RRKKEAAAKI	-1.29	Non-Toxin	-0.6	-1.43	1.47	4	1170.55
RKKEAAAKIL	-1.34	Non-Toxin	-0.37	-0.6	0.99	3	1127.53

KKEAAAKILM	-1.38	Non-Toxin	-0.17	0.04	0.56	2	1102.54
KEAAAKILMT	-1.5	Non-Toxin	-0.07	0.36	0.22	1	1075.47
EAAAKILMTA	-1.53	Non-Toxin	0.06	0.93	-0.13	0	1018.37
AAAKILMTAR	-1.37	Non-Toxin	-0.05	0.83	-0.13	2	1045.44
AAKILMTART	-1.27	Non-Toxin	-0.1	0.58	-0.12	2	1075.47
AKILMTARTV	-1.12	Non-Toxin	-0.07	0.82	-0.22	2	1103.53
KILMTARTVY	-1.58	Non-Toxin	-0.09	0.51	-0.4	2	1195.63
ILMTARTVYG	-1.31	Non-Toxin	0.04	0.86	-0.7	1	1124.51
LMTARTVYGP	-1.17	Non-Toxin	-0.04	0.25	-0.52	1	1108.46
MTARTVYGPG	-1.18	Non-Toxin	-0.08	-0.17	-0.34	1	1052.35
TARTVYGPGP	-0.81	Non-Toxin	-0.11	-0.52	-0.21	1	1018.27
ARTVYGPGPG	-0.79	Non-Toxin	-0.08	-0.49	-0.17	1	974.22
RTVYGPGPGT	-0.69	Non-Toxin	-0.12	-0.74	-0.16	1	1004.25
TVYGPGPGTM	-0.5	Non-Toxin	0.08	-0.1	-0.59	0	979.26
VYGPGPGTML	-0.66	Non-Toxin	0.15	0.35	-0.73	0	991.32
YGPGPGTMLF	-0.58	Non-Toxin	0.16	0.21	-0.83	0	1039.36
GPGPGTMLFT	-0.62	Non-Toxin	0.14	0.27	-0.64	0	977.29
PGPGTMLFTM	-0.58	Non-Toxin	0.15	0.5	-0.77	0	1051.43
GPGTMLFTML	-0.74	Non-Toxin	0.21	1.04	-0.95	0	1067.48
PGTMLFTMLR	-0.83	Non-Toxin	0.02	0.63	-0.65	1	1166.61
GTMLFTMLRK	-0.77	Non-Toxin	-0.09	0.4	-0.35	2	1197.67
TMLFTMLRKG	-0.71	Non-Toxin	-0.09	0.4	-0.35	2	1197.67
MLFTMLRKGP	-0.94	Non-Toxin	-0.08	0.31	-0.31	2	1193.68
LFTMLRKGPG	-0.78	Non-Toxin	-0.09	0.08	-0.18	2	1119.54
FTMLRKGPGP	-0.63	Non-Toxin	-0.15	-0.46	0	2	1103.49
TMLRKGPGPG	-0.63	Non-Toxin	-0.19	-0.78	0.25	2	1013.37
MLRKGPGPGY	-0.84	Non-Toxin	-0.17	-0.84	0.06	2	1075.44
LRKGPGPGYL	-0.76	Non-Toxin	-0.14	-0.65	0.01	2	1057.41
RKGPGPGYLN	-0.69	Non-Toxin	-0.26	-1.38	0.21	2	1058.35
KGPGPGYLNT	-0.68	Non-Toxin	-0.1	-1	-0.13	1	1003.27
GPGPGYLNTL	-0.79	Non-Toxin	0.06	-0.23	-0.61	0	988.26
PGPGYLNTLT	-1.06	Non-Toxin	0.03	-0.26	-0.65	0	1032.31
GPGYLNTLTL	-1.18	Non-Toxin	0.09	0.28	-0.83	0	1048.36
PGYLNTLTLA	-1.33	Non-Toxin	0.1	0.5	-0.88	0	1062.38
GYLNTLTLAV	-1.31	Non-Toxin	0.16	1.08	-1.03	0	1064.4
YLNTLTLAVE	-1.47	Non-Toxin	0.08	0.77	-0.73	-1	1136.46
LNTLTLAVEA	-1.49	Non-Toxin	0.1	1.08	-0.55	-1	1044.36
NTLTLAVEAA	-1.44	Non-Toxin	0.07	0.88	-0.42	-1	1002.27
TLTLAVEAAA	-1.39	Non-Toxin	0.16	1.41	-0.49	-1	959.24
LTLAVEAAAK	-1.24	Non-Toxin	0.07	1.09	-0.15	0	986.31
TLAVEAAAKW	-1.15	Non-Toxin	0.05	0.62	-0.31	0	1059.36
LAVEAAAKWS	-1.13	Non-Toxin	0.05	0.61	-0.24	0	1045.33
AVEAAAKWSM	-0.98	Non-Toxin	0.02	0.42	-0.19	0	1063.36
VEAAAKWSMA	-0.97	Non-Toxin	0.02	0.42	-0.19	0	1063.36
EAAAKWSMAT	-0.97	Non-Toxin	-0.05	-0.07	-0.08	0	1065.33
AAAKWSMATY	-0.78	Non-Toxin	0.01	0.15	-0.61	1	1099.39
AAKWSMATYY	-0.52	Non-Toxin	-0.01	-0.16	-0.79	1	1191.49

AKWSMATYYL	-0.54	Non-Toxin	0.02	0.04	-0.92	1	1233.58
KWSMATYYLF	-0.72	Non-Toxin	0.05	0.14	-1.12	1	1309.68
WSMATYYLFA	-0.98	Non-Toxin	0.19	0.71	-1.47	0	1252.58
SMATYYLFAA	-1.02	Non-Toxin	0.17	0.98	-1.18	0	1137.44
MATYYLFAAG	-1.21	Non-Toxin	0.22	1.02	-1.21	0	1107.42
ATYYLFAAGY	-0.96	Non-Toxin	0.19	0.7	-1.31	0	1139.4
TYYLFAAGYV	-1.07	Non-Toxin	0.22	0.94	-1.41	0	1167.46
YYLFAAGYVF	-1.16	Non-Toxin	0.3	1.29	-1.62	0	1213.53
YLFAAGYVFC	-0.97	Non-Toxin	0.3	1.67	-1.49	0	1153.49
LFAAGYVFCT	-0.8	Non-Toxin	0.28	1.73	-1.3	0	1091.42
FAAGYVFCTV	-0.84	Non-Toxin	0.28	1.77	-1.27	0	1077.39
AAGYVFCTVN	-0.83	Non-Toxin	0.16	1.14	-1	0	1044.32
AGYVFCTVNA	-0.54	Non-Toxin	0.16	1.14	-1	0	1044.32
GYVFCTVNAL	-0.46	Non-Toxin	0.19	1.34	-1.13	0	1086.41
YVFCTVNALA	-0.69	Non-Toxin	0.2	1.56	-1.18	0	1100.43
VFCTVNALAA	-0.7	Non-Toxin	0.22	1.87	-1	0	1008.33
FCTVNALAAG	-0.68	Non-Toxin	0.18	1.41	-0.85	0	966.25
CTVNALAAGL	-0.94	Non-Toxin	0.17	1.51	-0.78	0	932.24
TVNALAAGLM	-1.05	Non-Toxin	0.2	1.45	-0.81	0	960.3
VNALAAGLMI	-0.98	Non-Toxin	0.29	1.97	-0.95	0	972.36
NALAAGLMIE	-0.83	Non-Toxin	0.17	1.2	-0.5	-1	1002.34
ALAAGLMIER	-1.32	Non-Toxin	0.06	1.1	-0.22	0	1044.42
LAAGLMIERF	-1.44	Non-Toxin	0.09	1.2	-0.42	0	1120.52
AAGLMIERFV	-1.39	Non-Toxin	0.09	1.24	-0.39	0	1106.49
AGLMIERFVS	-1.39	Non-Toxin	0.04	0.98	-0.31	0	1122.49
GLMIERFVSL	-1.51	Non-Toxin	0.07	1.18	-0.44	0	1164.58
LMIERFVSLA	-1.61	Non-Toxin	0.08	1.4	-0.49	0	1178.6
MIERFVSLAA	-1.63	Non-Toxin	0.05	1.2	-0.36	0	1136.51
IERFVSLAAG	-1.52	Non-Toxin	0.04	0.97	-0.23	0	1062.37
ERFVSLAAGF	-1.64	Non-Toxin	0.03	0.8	-0.3	0	1096.38
RFVSLAAGFE	-1.32	Non-Toxin	0.03	0.8	-0.3	0	1096.38
FVSLAAGFEL	-1.5	Non-Toxin	0.26	1.63	-0.78	-1	1053.36
VSLAAGFELE	-1.46	Non-Toxin	0.14	1	-0.23	-2	1035.3
SLAAGFELED	-1.24	Non-Toxin	0.01	0.23	0.22	-3	1051.25
LAAGFELEDF	-1.48	Non-Toxin	0.1	0.59	-0.06	-3	1111.35
AAGFELEDFI	-1.35	Non-Toxin	0.12	0.66	-0.06	-3	1111.35
AGFELEDFIP	-1.22	Non-Toxin	0.09	0.32	-0.01	-3	1137.39
GFELEDFIPM	-1.04	Non-Toxin	0.09	0.33	-0.09	-3	1197.51
FELEDFIPMA	-1.18	Non-Toxin	0.1	0.55	-0.14	-3	1211.53
ELEDFIPMAA	-1.24	Non-Toxin	0.06	0.45	0.06	-3	1135.43
LEDFIPMAAG	-1.15	Non-Toxin	0.14	0.76	-0.24	-2	1063.37
EDFIPMAAGM	-1.08	Non-Toxin	0.11	0.57	-0.19	-2	1081.4
DFIPMAAGMA	-1.17	Non-Toxin	0.2	1.1	-0.54	-1	1023.36
FIPMAAGMAS	-0.98	Non-Toxin	0.24	1.37	-0.81	0	995.35
IPMAAGMASL	-0.96	Non-Toxin	0.24	1.47	-0.74	0	961.34
PMAAGMASLV	-0.95	Non-Toxin	0.22	1.44	-0.71	0	947.31
MAAGMASLVL	-1.13	Non-Toxin	0.28	1.98	-0.89	0	963.36

AAGMASLVLA	-1.06	Non-Toxin	0.28	1.97	-0.81	0	903.24
AGMASLVLAR	-1.08	Non-Toxin	0.07	1.34	-0.46	1	988.35
GMASLVLARK	-1.17	Non-Toxin	-0.06	0.77	-0.11	2	1045.45
MASLVLARKA	-1.23	Non-Toxin	-0.05	0.99	-0.16	2	1059.47
ASLVLARKAA	-1.11	Non-Toxin	-0.05	0.98	-0.08	2	999.35
SLVLARKAAG	-1.16	Non-Toxin	-0.06	0.76	-0.03	2	985.33
LVLARKAAGC	-0.98	Non-Toxin	-0.03	1.09	-0.16	2	1001.39
VLARKAAGCQ	-0.54	Non-Toxin	-0.15	0.36	0.04	2	1016.36
LARKAAGCQY	-0.5	Non-Toxin	-0.21	-0.19	-0.04	2	1080.4
ARKAAGCQYL	-0.51	Non-Toxin	-0.21	-0.19	-0.04	2	1080.4
RKAAGCQYLN	-0.56	Non-Toxin	-0.29	-0.72	0.03	2	1123.43
KAAGCQYLNT	-0.54	Non-Toxin	-0.14	-0.34	-0.31	1	1068.35
AAGCQYLNTL	-0.72	Non-Toxin	0.03	0.43	-0.79	0	1053.34
AGCQYLNTLT	-0.72	Non-Toxin	-0.02	0.18	-0.78	0	1083.37
GCQYLNTLTL	-0.86	Non-Toxin	0.01	0.38	-0.91	0	1125.46
CQYLNTLTLA	-1.19	Non-Toxin	0.02	0.6	-0.96	0	1139.48
QYLNTLTLAA	-1.46	Non-Toxin	0.04	0.53	-0.91	0	1107.42
YLNTLTLAAG	-1.48	Non-Toxin	0.13	0.84	-0.93	0	1036.34
LNTLTLAAGF	-1.43	Non-Toxin	0.19	1.25	-0.95	0	1020.34
NTLTLAAGFS	-1.42	Non-Toxin	0.11	0.79	-0.74	0	994.25
TLTLAAGFSY	-1.48	Non-Toxin	0.17	1.01	-0.99	0	1043.32
LTLAAGFSYF	-1.44	Non-Toxin	0.25	1.36	-1.2	0	1089.39
TLAAGFSYFA	-1.5	Non-Toxin	0.22	1.16	-1.07	0	1047.3
LAAGFSYFAV	-1.41	Non-Toxin	0.3	1.65	-1.18	0	1045.33
AAGFSYFAVH	-1.29	Non-Toxin	0.2	0.95	-1.05	0.5	1069.31
AGFSYFAVHF	-1.31	Non-Toxin	0.24	1.05	-1.25	0.5	1145.41
GFSYFAVHFI	-1.29	Non-Toxin	0.29	1.32	-1.38	0.5	1187.5

Table S6. VC evaluation for possible CTL epitopes that will be generated and homology of new epitopes with human peptides.

Start	Peptide	HLA alleles	Human proteins	human peptides
0	GIINTLQKY	HLA-A*29:01, HLA-B*15:01, HLA-B*15:12	similar with human beta defensin	^{33}GIINTLQKY^{41}
1	IINTLQKYY	HLA-A*29:01	similar with human beta defensin	^{34}IINTLQKYY^{42}
3	NTLQKYYCR	HLA-A*33:03	similar with human beta defensin	^{4}NTLQKYYCR^{12}
4	TLQKYYCRV	HLA-A*02:03	similar with human beta defensin	^{37}TLQKYYCRV^{45}
11	RVRGGRCAV	HLA-A*30:01, HLA-B*07:02, HLA-B*07:05, HLA-B*56:01,	similar with human beta defensin	^{44}RVRGGRCAV^{52}
12	VRGGRCAVL	HLA-B*27:06	similar with human beta defensin	^{45}VRGGRCAVL^{53}
15	GRCAVLSCL	HLA-B*27:06	similar with human beta defensin	^{48}GRCAVLSCL^{56}
46	AAAKKSAFY	HLA-A*01:01, HLA-B*15:12,	Mitochondrial ribosomal protein L1	^{22}AAAKKSA^{28}
47	AAKKSAFYI	HLA-A*30:01		
51	SAFYILPSI	HLA-A*32:01, HLA-B*13:02, HLA-B*51:01, HLA-B*51:02, HLA-B*52:01		
52	AFYILPSII	HLA-B*52:01		
56	LPSIISNEK	HLA-B*56:01		
94	SLRPDTRYV	HLA-A*02:03		
95	LRPDTRYVL	HLA-B*27:06		
96	RPDTRYVLM	HLA-B*07:02, HLA-B*07:05,		
103	LMDGPGPGV	HLA-A*02:01	immunoglobulin heavy chain junction region	4DGPGPGV10
110	GVSTQEFRY	HLA-A*01:01, HLA-A*29:01,		
116	FRYMNSQGL	HLA-B*15:10, HLA-B*27:06, HLA-B*38:02		
117	RYMNSQGLL	HLA-A*24:02, HLA-A*24:07, HLA-A*24:10		
125	LEAAAKWSM	HLA-B*18:01, HLA-B*18:02, HLA-B*40:01, HLA-B*44:03,		
128	AAKWSMATY	HLA-A*30:01, HLA-B*15:01, HLA-B*15:02, HLA-B*15:12, HLA-B*15:13, HLA-B*15:17, HLA-B*15:21, HLA-B*15:25, HLA-B*15:32, HLA-B*35:05, HLA-B*35:30, HLA-B*46:01, HLA-B*57:01		
130	KWSMATYYL	HLA-A*24:02, HLA-A*24:07, HLA-A*24:10		
131	WSMATYYLF*	HLA-A*24:02, HLA-A*24:07, HLA-A*24:10, HLA-B*15:13, HLA-B*15:17, HLA-B*57:01, HLA-B*58:01		
133	MATYYLFAA	HLA-B*56:01		
135	TYYLFAAGY	HLA-A*29:01		
136	YYLFAAGYV	HLA-A*24:10		
137	YLFAAGYVF	HLA-A*02:01, HLA-A*02:06, HLA-A*02:07, HLA-A*24:02, HLA-A*24:07, HLA-A*29:01, HLA-A*32:01, HLA-B*13:01, HLA-B*15:01, HLA-B*15:02, HLA-B*15:10, HLA-B*15:12, HLA-B*15:13, HLA-B*15:21, HLA-B*15:25, HLA-B*15:32, HLA-B*18:01, HLA-B*18:02, HLA-B*35:01, HLA-B*35:02, HLA-B*35:05, HLA-B*35:30, HLA-B*38:02, HLA-B*46:01, HLA-B*48:01, HLA-B*56:01, HLA-B*56:02	immunoglobulin light chain junction region	8FAAGYVF14
143	YVFCTVNAL*	HLA-A*02:01, HLA-A*02:03, HLA-A*02:06, HLA-A*02:07, HLA-A*26:01, HLA-A*34:01, HLA-B*07:02, HLA-B*07:05, HLA-B*15:02, HLA-B*15:10, HLA-B*15:21, HLA-B*35:01, HLA-B*35:02, HLA-B*35:05, HLA-B*35:30, HLA-B*38:02, HLA-B*46:01, HLA-B*48:01, HLA-B*56:01, HLA-B*56:02,		
155	LMIERFVSL*	HLA-A*02:01, HLA-A*02:03, HLA-A*02:06, HLA-A*02:07, HLA-A*32:01, HLA-B*08:01, HLA-B*15:01, HLA-B*15:02, HLA-B*15:10, HLA-B*15:12, HLA-B*15:21, HLA-B*15:25, HLA-B*15:32, HLA-B*35:02, HLA-B*37:01, HLA-B*38:02, HLA-B*46:01, HLA-B*48:01,		
157	IERFVSLAA	HLA-B*40:06, HLA-B*41:01,		
159	RFVSLAAGF	HLA-A*24:02, HLA-A*24:10,	hCG2019424	52VSLAAGF58
161	VSLAAGFEL	HLA-B*58:01	hCG2019424	52VSLAAGF58

167	FELEDFIPM*	HLA-B*13:01, HLA-B*13:02, HLA-B*15:10, HLA-B*18:01, HLA-B*18:02, HLA-B*35:02, HLA-B*37:01, HLA-B*38:02, HLA-B*40:01, HLA-B*40:02, HLA-B*40:06, HLA-B*41:01, HLA-B*44:03, HLA-B*48:01,
175	MAAGMASLV	HLA-B*56:01, HLA-B*56:07,
178	GMASLVLAR	HLA-A*74:01
179	MASLVLARK*	HLA-A*03:01, HLA-A*11:01, HLA-A*11:04, HLA-A*30:01, HLA-A*33:03, HLA-A*34:01, HLA-A*74:01
186	RKAAGCQYL	HLA-B*27:06, HLA-B*48:01,
191	CQYLNTLTL*	HLA-B*13:01, HLA-B*13:02, HLA-B*15:10, HLA-B*27:06, HLA-B*37:01, HLA-B*38:02, HLA-B*48:01, HLA-B*52:01,
193	YLNTLTLAA	HLA-A*02:03
197	LTLAAGFSY	HLA-A*01:01, HLA-A*11:01, HLA-A*29:01, HLA-B*15:01, HLA-B*15:02, HLA-B*15:12, HLA-B*15:13, HLA-B*15:17, HLA-B*15:21, HLA-B*15:25, HLA-B*35:01, HLA-B*35:05, HLA-B*35:30, HLA-B*46:01, HLA-B*57:01, HLA-B*58:01,
198	TLAAGFSYF	HLA-A*26:01, HLA-A*29:01, HLA-A*32:01, HLA-A*34:01, HLA-B*15:01, HLA-B*15:02, HLA-B*15:12, HLA-B*15:13, HLA-B*15:21, HLA-B*15:25, HLA-B*15:32, HLA-B*46:01,
202	GFSYFAVHF	HLA-A*24:02, HLA-A*24:10,
203	FSYFAVHFI*	HLA-B*13:02, HLA-B*51:01, HLA-B*51:02, HLA-B*52:01,

*CTL epitopes originally put in the vaccine construct

Table S7. VC evaluation for possible HTL epitopes that will be generated and possibility of new epitopes homology with human peptides.

Pos	15-mer peptide	Of	Core peptide	HLA alleles
48	AAKKSAFYILPSIIS	6	FYILPSIIS	DRB1*04:05, DRB1*10:01, DRB1*16:02
49	AKKSAFYILPSIISN	5	FYILPSIIS	DRB1*01:01, DRB1*04:01, DRB1*04:03, DRB1*04:05, DRB1*04:06, DRB1*08:03, DRB1*10:01, DRB1*11:01, DRB1*12:02, DRB1*14:07, DRB1*15:02, DRB1*16:02,
50	KKSAFYILPSIISNE	4	FYILPSIIS	DRB1*01:01, DRB1*04:01, DRB1*04:02, DRB1*04:03, DRB1*04:05, DRB1*04:06, DRB1*07:01, DRB1*08:03, DRB1*09:01, DRB1*10:01, DRB1*11:01, DRB1*12:02, DRB1*14:07, DRB1*15:01, DRB1*15:02, DRB1*16:02,
51	KSAFYILPSIISNEK*	3	FYILPSIIS	DRB1*01:01, DRB1*04:01, DRB1*04:02, DRB1*04:03, DRB1*04:05, DRB1*04:06, DRB1*07:01, DRB1*08:03, DRB1*09:01, DRB1*10:01, DRB1*11:01, DRB1*12:02, DRB1*13:02, DRB1*14:04, DRB1*1405, DRB1*14:07, DRB1*15:01, DRB1*15:02, DRB1*16:02
52	SAFYILPSIISNEKG	2	FYILPSIIS	DRB1*01:01, DRB1*04:01, DRB1*04:03, DRB1*04:05, DRB1*04:06, DRB1*08:03, DRB1*10:01, DRB1*11:01, DRB1*12:02, DRB1*14:07, DRB1*15:02, DRB1*16:02,
53	AFYILPSIISNEKGP	1	FYILPSIIS	DRB1*01:01, DRB1*10:01, DRB1*16:02
55	YILPSIISNEKGPGP	5	IISNEKGPG	DRB1*0301, DRB1*11:01, DRB1*13:02, DRB1*1401, DRB1*14:04, DRB1*1405, DRB1*14:07, DRB1*1454,
56	ILPSIISNEKGPGPG	4	IISNEKGPG	DRB1*0301, DRB1*11:01, DRB1*13:02, DRB1*1401, DRB1*14:04, DRB1*1405, DRB1*14:07, DRB1*1454,
57	LPSIISNEKGPGPGG	3	IISNEKGPG	DRB1*0301, DRB1*11:01, DRB1*13:02, DRB1*1401, DRB1*14:04, DRB1*1405, DRB1*14:07, DRB1*1454,
58	PSIISNEKGPGPGGR	2	IISNEKGPG	DRB1*0301, DRB1*11:01, DRB1*13:02, DRB1*1401, DRB1*14:04, DRB1*1405, DRB1*14:07, DRB1*1454,
68	GPGGRLIIRENNRVV	5	LIIRENNRV	DRB1*04:02, DRB1*13:02, DRB1*14:04, DRB1*15:01, DRB1*15:02
69	PGGRLIIRENNRVVI	4	LIIRENNRV	DRB1*04:02, DRB1*08:03, DRB1*13:02, DRB1*1401, DRB1*14:04, DRB1*1405, DRB1*1454, DRB1*15:01, DRB1*15:02, DRB1*16:02,
70	GGRLIIRENNRVVIS	5	IRENNRVVI	DRB1*0301, DRB1*04:02, DRB1*08:03, DRB1*12:02, DRB1*13:02, DRB1*1401, DRB1*14:04, DRB1*1405, DRB1*14:07, DRB1*1454, DRB1*15:01, DRB1*15:02, DRB1*16:02
71	GRLIIRENNRVVISS*	4	IRENNRVVI	DRB1*0301, DRB1*04:02, DRB1*12:02, DRB1*13:02, DRB1*1401, DRB1*14:04, DRB1*1405, DRB1*14:07, DRB1*1454, DRB1*15:01, DRB1*15:02, DRB1*16:02,
72	RLIIRENNRVVISSD*	3	IRENNRVVI	DRB1*0301, DRB1*04:02, DRB1*13:02, DRB1*1401, DRB1*14:04, DRB1*1405, DRB1*14:07, DRB1*1454, DRB1*15:01, DRB1*15:02,
73	LIIRENNRVVISSDG	2	IRENNRVVI	DRB1*13:02, DRB1*1401, DRB1*1405, DRB1*14:07, DRB1*1454
74	IIRENNRVVISSDGP	1	IRENNRVVI	DRB1*13:02
76	RENNRVVISSDGPGP	5	VVISSDGPG**	DRB1*04:01, DRB1*04:02, DRB1*04:03, DRB1*04:06,
77	ENNRVVISSDGPGPG	4	VVISSDGPG**	DRB1*04:01, DRB1*04:02, DRB1*04:03, DRB1*04:05, DRB1*04:06
78	NNRVVISSDGPGPGA	5	ISSDGPGPG	DRB1*0301, DRB1*04:01, DRB1*04:02, DRB1*04:03, DRB1*04:05, DRB1*04:06,
79	NRVVISSDGPGPGAY	4	ISSDGPGPG	DRB1*0301, DRB1*04:01, DRB1*04:02, DRB1*04:03, DRB1*04:06
80	RVVISSDGPGPGAYE	3	ISSDGPGPG	DRB1*0301, DRB1*04:01,
89	GPGAYESLRPDTRYV	4	YESLRPDTR	DRB1*01:01
90	PGAYESLRPDTRYVL	3	YESLRPDTR	DRB1*01:01, DRB1*0301, DRB1*1401, DRB1*1454,
91	GAYESLRPDTRYVLM	5	LRPDTRYVL	DRB1*0301, DRB1*13:02, DRB1*1401, DRB1*14:04, DRB1*1405, DRB1*14:07, DRB1*1454
92	AYESLRPDTRYVLMD*	4	LRPDTRYVL	DRB1*0301, DRB1*13:02, DRB1*1401, DRB1*14:04, DRB1*1405, DRB1*14:07, DRB1*1454
93	YESLRPDTRYVLMDG	3	LRPDTRYVL	DRB1*0301, DRB1*13:02, DRB1*1401, DRB1*14:04, DRB1*1405, DRB1*14:07, DRB1*1454
94	ESLRPDTRYVLMDGP	2	LRPDTRYVL	DRB1*0301, DRB1*13:02, DRB1*1401, DRB1*14:04, DRB1*1405, DRB1*14:07, DRB1*1454
95	SLRPDTRYVLMDGPG	1	LRPDTRYVL	DRB1*0301
97	RPDTRYVLMDGPGPG	5	YVLMDGPGP	DRB1*01:01, DRB1*10:01, DRB1*16:02
98	PDTRYVLMDGPGPGV	4	YVLMDGPGP	DRB1*01:01, DRB1*04:01, DRB1*10:01, DRB1*16:02,
99	DTRYVLMDGPGPGVS	3	YVLMDGPGP	DRB1*01:01, DRB1*0301, DRB1*04:01, DRB1*10:01, DRB1*16:02
100	TRYVLMDGPGPGVST	2	YVLMDGPGP	DRB1*01:01, DRB1*0301,
111	GVSTQEFRYMNSQGL	6	FRYMNSQGL	DRB1*01:01, DRB1*07:01, DRB1*08:03, DRB1*09:01, DRB1*10:01, DRB1*15:02, DRB1*16:02

112	VSTQEFRYMNSQGLL*	5	FRYMNSQGL	DRB1*01:01, DRB1*04:01, DRB1*04:05, DRB1*07:01, DRB1*08:03, DRB1*09:01, DRB1*10:01, DRB1*12:02, DRB1*13:02, DRB1*14:07, DRB1*15:01, DRB1*15:02, DRB1*16:02
113	STQEFRYMNSQGLLE	4	FRYMNSQGL	DRB1*01:01, DRB1*04:01, DRB1*04:02, DRB1*04:03, DRB1*04:05, DRB1*04:06, DRB1*07:01, DRB1*08:03, DRB1*09:01, DRB1*10:01, DRB1*11:01, DRB1*12:02, DRB1*13:02, DRB1*1401, DRB1*14:04, DRB1*14:07, DRB1*1454, DRB1*15:01, DRB1*15:02, DRB1*16:02,
114	TQEFRYMNSQGLLEA	3	FRYMNSQGL	DRB1*01:01, DRB1*04:01, DRB1*04:02, DRB1*04:03, DRB1*04:05, DRB1*04:06, DRB1*07:01, DRB1*08:03, DRB1*09:01, DRB1*10:01, DRB1*11:01, DRB1*12:02, DRB1*13:02, DRB1*1401, DRB1*14:04, DRB1*1405, DRB1*14:07, DRB1*1454, DRB1*15:01, DRB1*15:02, DRB1*16:02
115	QEFRYMNSQGLLEAA	2	FRYMNSQGL	DRB1*01:01, DRB1*04:01, DRB1*04:05, DRB1*07:01, DRB1*08:03, DRB1*09:01, DRB1*10:01, DRB1*12:02, DRB1*13:02, DRB1*14:07, DRB1*15:01, DRB1*15:02, DRB1*16:02
116	EFRYMNSQGLLEAAA	1	FRYMNSQGL	DRB1*01:01, DRB1*07:01, DRB1*09:01, DRB1*10:01, DRB1*15:02, DRB1*16:02,
121	NSQGLLEAAAKWSMA	5	LEAAAKWSM	DRB1*1401, DRB1*14:04, DRB1*1405, DRB1*1454,
122	SQGLLEAAAKWSMAT	4	LEAAAKWSM	DRB1*1401, DRB1*14:04, DRB1*1405, DRB1*1454,
123	QGLLEAAAKWSMATY	3	LEAAAKWSM	DRB1*1401, DRB1*14:04, DRB1*1405, DRB1*1454,
139	LFAAGYVFCTVNALA	5	YVFCTVNAL	DRB1*09:01
140	FAAGYVFCTVNALAA	4	YVFCTVNAL	DRB1*01:01, DRB1*07:01, DRB1*09:01, DRB1*10:01, DRB1*14:07
141	AAGYVFCTVNALAAG	3	YVFCTVNAL	DRB1*01:01, DRB1*04:01, DRB1*07:01, DRB1*09:01, DRB1*10:01, DRB1*14:07, DRB1*16:02
142	AGYVFCTVNALAAGL	2	YVFCTVNAL	DRB1*01:01, DRB1*09:01, DRB1*10:01
153	AAGLMIERFVSLAAG	5	IERFVSLAA	DRB1*04:02, DRB1*15:01, DRB1*15:02
154	AGLMIERFVSLAAGF	4	IERFVSLAA	DRB1*04:02, DRB1*15:01, DRB1*15:02
155	GLMIERFVSLAAGFE	3	IERFVSLAA	DRB1*04:02, DRB1*15:01, DRB1*15:02, DRB1*16:02,
156	LMIERFVSLAAGFEL	5	FVSLAAGFE**	DRB1*04:05, DRB1*15:01, DRB1*15:02
157	MIERFVSLAAGFELE	4	FVSLAAGFE**	DRB1*04:05, DRB1*09:01, DRB1*10:01
158	IERFVSLAAGFELED	3	FVSLAAGFE**	DRB1*01:01, DRB1*04:03, DRB1*04:05, DRB1*04:06, DRB1*07:01, DRB1*08:03, DRB1*09:01, DRB1*10:01, DRB1*16:02
159	ERFVSLAAGFELEDF	2	FVSLAAGFE**	DRB1*01:01, DRB1*04:05, DRB1*09:01, DRB1*10:01,
168	FELEDFIPMAAGMAS	5	FIPMAAGMA	DRB1*01:01, DRB1*10:01, DRB1*11:01, DRB1*16:02,
169	ELEDFIPMAAGMASL	4	FIPMAAGMA	DRB1*01:01, DRB1*04:01, DRB1*10:01, DRB1*11:01, DRB1*16:02
170	LEDFIPMAAGMASLV	3	FIPMAAGMA	DRB1*01:01, DRB1*04:01, DRB1*10:01, DRB1*11:01, DRB1*16:02
171	EDFIPMAAGMASLVL	2	FIPMAAGMA	DRB1*01:01, DRB1*10:01,
177	AAGMASLVLARKAAG	6	LVLARKAAG	DRB1*11:01, DRB1*1405,
178	AGMASLVLARKAAGC	5	LVLARKAAG	DRB1*11:01, DRB1*1405,
179	GMASLVLARKAAGCQ	4	LVLARKAAG	DRB1*11:01, DRB1*1401, DRB1*1405, DRB1*1454,
180	MASLVLARKAAGCQY	3	LVLARKAAG	DRB1*11:01, DRB1*1401, DRB1*1405, DRB1*1454,
181	ASLVLARKAAGCQYL	2	LVLARKAAG	DRB1*11:01, DRB1*1405,
190	AGCQYLNTLTLAAGF	4	YLNTLTLAA	DRB1*04:01
191	GCQYLNTLTLAAGFS	3	YLNTLTLAA	DRB1*04:01

*HTL epitopes originally put in the vaccine construct

**HTL epitopes with core peptides sequence homologous to human peptides. Peptide FVSLAAGFE contain residue that match with heptamer VSLAAGF from human protein hCG2019424 (sequence ID EAX10398.1). Peptide VVISSDGPG contain residues that match with heptamer VVISSDG from guanine nucleotide binding protein (G protein) (sequence ID EAW53700.1).

Table S8. Non homology analysis of VC peptides against gut microbiota proteomes using PBIT server. All 43 of 9-mer CTL epitopes (left panel) and 17 of 9-mer core peptides of HTL epitopes (right panel) that were generated from VC were input into the PBIT server, which will calculate the similarity of peptide sequences with the sequences from the gut microbiomes.

Non-homology analysis against gut microbiota proteomes

Number of input sequence/s:43

Criteria for non-homologous proteins: E-value > 0.005 or % sequence identity < 50

☐	Query name	No. of hits	BLAST report
☐	1	Undefined	Download
☐	2	Undefined	Download
☐	3	Undefined	Download
☐	4	Undefined	Download
☐	5	Undefined	Download
☐	6	Undefined	Download
☐	7	Undefined	Download
☐	8	Undefined	Download
☐	9	Undefined	Download
☐	10	Undefined	Download
☐	11	Undefined	Download
☐	12	Undefined	Download
☐	13	Undefined	Download
☐	14	Undefined	Download
☐	15	Undefined	Download
☐	16	Undefined	Download
☐	17	Undefined	Download
☐	18	Undefined	Download
☐	19	Undefined	Download
☐	20	Undefined	Download
☐	21	Undefined	Download
☐	22	Undefined	Download
☐	23	Undefined	Download
☐	24	Undefined	Download
☐	25	Undefined	Download
☐	26	Undefined	Download
☐	27	Undefined	Download
☐	28	Undefined	Download
☐	29	Undefined	Download
☐	30	Undefined	Download
☐	31	Undefined	Download
☐	32	Undefined	Download
☐	33	Undefined	Download
☐	34	Undefined	Download
☐	35	Undefined	Download
☐	36	Undefined	Download
☐	37	Undefined	Download
☐	38	Undefined	Download
☐	39	Undefined	Download
☐	40	Undefined	Download
☐	41	Undefined	Download
☐	42	Undefined	Download
☐	43	Undefined	Download

43 of the 43 input sequences are non-homologous (highlighted in blue) **based on input BLAST parameters**.

Non-homology analysis against gut microbiota proteomes

Number of input sequence/s:17

Criteria for non-homologous proteins: E-value > 0.005 or % sequence identity < 50

☐	Query name	No. of hits	BLAST report
☐	1	Undefined	Download
☐	2	Undefined	Download
☐	3	Undefined	Download
☐	4	Undefined	Download
☐	5	Undefined	Download
☐	6	Undefined	Download
☐	7	Undefined	Download
☐	8	Undefined	Download
☐	9	Undefined	Download
☐	10	Undefined	Download
☐	11	Undefined	Download
☐	12	Undefined	Download
☐	13	Undefined	Download
☐	14	Undefined	Download
☐	15	Undefined	Download
☐	16	Undefined	Download
☐	17	Undefined	Download

17 of the 17 input sequences are non-homologous (highlighted in blue) **based on input BLAST parameters**.

Table S9. Summary of the Top 10 models generated by HDOCK for interaction between TLR4 and VC.

Rank	1	2	3	4	5	6	7	8	9	10
Docking Score	-283.87	-247.70	-247.20	-246.91	-246.37	-242.17	-240.76	-239.35	-239.05	-238.82
Ligand rmsd (Å)	50.26	45.64	51.23	53.39	34.65	44.80	43.67	56.62	44.74	29.67
Interface residues	Model 1	Model 2	Model 3	Model 4	Model 5	Model 6	Model 7	Model 8	Model 9	Model 10

Table S10. Receptor-ligand interface residues pair for Model 1.

Receptor interface residue(s)	Ligand interface residue(s):	Receptor-ligand interface residue pair(s):
ASN 205A 2.788	GLY 1A 2.593	205A - 21A 2.788
HIS 229A 2.353	ILE 2A 4.741	229A - 20A 4.653
HIS 256A 3.052	ILE 3A 3.178	229A - 21A 2.353
GLU 286A 3.344	THR 5A 3.357	256A - 21A 4.576
GLU 287A 2.934	GLN 7A 2.599	256A - 38A 3.052
ASN 309A 3.135	LYS 8A 1.779	286A - 35A 4.823
VAL 310A 4.100	TYR 9A 2.827	286A - 36A 3.344
SER 311A 3.307	TYR 10A 3.110	287A - 35A 3.486
GLN 333A 2.498	ARG 17A 3.329	287A - 38A 2.934
HIS 334A 3.489	VAL 20A 4.653	309A - 36A 3.135
LYS 354A 4.414	LEU 21A 2.353	310A - 36A 4.100
ARG 355A 2.827	SER 34A 2.498	311A - 34A 4.045
GLU 376A 1.779	THR 35A 3.486	311A - 35A 4.339
PHE 377A 2.840	ARG 36A 3.135	311A - 36A 3.307
LYS 402A 3.318	ARG 38A 2.934	333A - 34A 2.498
TYR 403A 3.591		333A - 35A 4.826
GLU 425A 2.599		333A - 36A 3.871
HIS 426A 3.445		334A - 34A 3.489
ILE 450A 4.142		354A - 9A 4.414
TYR 451A 2.593		355A - 9A 2.827
VAL 475A 4.057		355A - 10A 3.584
LYS 477A 3.743		355A - 17A 3.329
PHE 500A 4.404		355A - 34A 4.369
		376A - 8A 1.779
		376A - 9A 2.931
		377A - 9A 2.840
		377A - 10A 3.110
		377A - 17A 4.836
		402A - 7A 3.318
		402A - 8A 4.316
		402A - 9A 3.657
		403A - 9A 3.908
		403A - 10A 3.591
		425A - 5A 3.357
		425A - 7A 2.599
		426A - 3A 3.445
		426A - 5A 4.930
		450A - 3A 4.142
		450A - 5A 4.837
		451A - 1A 2.593
		451A - 2A 4.741
		451A - 3A 3.178
		475A - 1A 4.554
		475A - 3A 4.057
		477A - 1A 3.743
		477A - 2A 4.797
		500A - 1A 4.404
